# The first detection of *Pneumocystis jirovecii* in asthmatic patients post-COVID-19 in Jordan

**DOI:** 10.17305/bjbms.2022.7335

**Published:** 2022-05-18

**Authors:** Ahmad R. Alsayed, Wamidh Talib, Abdullah Al-Dulaimi, Safa Daoud, Mohammed Al Maqbali

**Affiliations:** 1 Department of Clinical Pharmacy and Therapeutics, Faculty of Pharmacy, Applied Science Private University, Amman, Jordan; 2 Department of Pharmaceutical Chemistry and Pharmacognosy, Faculty of Pharmacy, Applied Science Private University, Amman, Jordan; 3 Department of Nursing Midwifery and Health, Northumbria University, Newcastle-Upon-Tyne, UK

**Keywords:** Asthma, COVID-19, developing country, *Pneumocystis jirovecii*, polymerase chain reaction

## Abstract

*Pneumocystis jirovecii* pneumonia (PCP), caused by fungal species named *P. jirovecii*, is a frequent opportunistic infection in those with human immunodeficiency virus infection. However, PCP has been documented in immunocompetent patients. This study aims to determine if *P. jirovecii* detection occurs in asthma patients following coronavirus disease 2019 (COVID-19) in a Jordanian cohort. Another aim was to evaluate a method of TaqMan quantitative polymerase chain reaction (qPCR) assay to detect P. jirovecii, from sputum samples. The nasopharyngeal swabs were used to detect SARS-CoV-2 and sputum samples were tested for *P. jirovecii* using real-time qPCR assay. Beta-tubulin (BT) and dihydrofolate reductase (DHFR) genes were the directed targets of *P. jirovecii*. The results showed that the mean qPCR efficiencies of BT and DHFR were 96.37% and 100.13%, respectively. Three out of 31 included patients (9.7%) had a positive *P. jirovecii*. All of the three patients had used oral corticosteroids (OCS) in the past 2 months due asthma exacerbation and were treated with OCS for COVID-19. This is the first study based in Jordan to demonstrate that *P. jirovecii* and COVID-19 can coexist and that it is important to maintain a broad differential diagnosis, especially in immunocompromised patients. Chronic lung disease can be a risk factor for the *P. jirovecii* colonization possibly due to corticosteroid’s immunosuppression.

## INTRODUCTION

A current global pandemic of coronavirus infectious disease 2019 (COVID-19) is caused by severe acute respiratory syndrome coronavirus 2 (SARS-CoV-2). Infection with this virus results in a wide range of symptoms, from asymptomatic illness to severe acute respiratory distress syndrome (ARDS), interstitial pneumonia associated with respiratory failure, and even death [1,2]. Comorbidities, including respiratory disease, were documented as contributing factors for more severe form of the disease and worse prognosis [3-5].

Other respiratory viruses, for example, seasonal/pandemic influenza exhibit varying degrees of coinfection with bacteria and fungi [6]. In addition, coinfection has been linked to more severe consequences during the pandemic [6].

While clinical care is primarily directed at diagnosing and managing COVID-19, there is emerging evidence that this disease can be complicated with developing concurrent pulmonary infections including fungal superinfection [7-10]. The coinfection with other pathogens can increase the disease symptoms and mortality, in addition to making the diagnosis and management more difficult [11]. As a result, there is a clinical need for rigorous examination of coinfection in COVID-19 patients as invasive fungal infections are not uncommon in COVID-19 patients [12].

*Pneumocystis jirovecii* pneumonia (PCP or PJP) caused by fungal species of *P. jirovecii* is a frequent opportunistic infection in those with human immunodeficiency virus (HIV) infection [8]. Although subclinical infection (colonization) with *P. jirovecii* reaches 9% in COVID-19 patients hospitalized in intensive care units (ICUs), only a few cases of PCP have been reported thus far, almost exclusively in immunocompromised patients [12-14]. However, PCP has been documented in immunocompetent individuals with COVID-19 [13-16].

Despite being not typically immunosuppression state, chronic lung disease can be a risk factor for the *P. jirovecii* colonization possibly as a result of structural lung destruction, corticosteroid’s immunosuppression, and smoking [17]. The previous studies based on animals propose an association between *P. jirovecii* infection and both of the obstructive pulmonary diseases; chronic obstructive lung disease (COPD); and asthma [18,19]. While oral corticosteroids (OCSs) are considered an independent PCP risk factor and usually used to treat asthma exacerbation, PCP is unusual in patients with asthma without any other identified risk factors [20].

At the moment, it is uncertain whether or not patients with asthma and COVID-19 are susceptible to *P. jirovecii* colonization in Jordan as there are lack of any regional publications about *P. jirovecii* and it is not tested routinely. Moreover, because most of the previous studies used nested PCR that associated with high chance of contamination, *P. jirovecii* prevalence might be overestimated in other countries. The purpose of this study was therefore to determine the prevalence of *P. jirovecii* coinfections in asthmatic patients with COVID-19 and to assess a method of TaqMan quantitative polymerase chain reaction (qPCR) assay in detecting *P. jirovecii*, from sputum samples.

## MATERIALS AND METHODS

### Study design and participants

Among asthmatic adult patients with confirmed COVID-19 not requiring hospitalization from April 1, 2021, to November 1, 2021, we analyzed all sputum samples for *P. jirovecii* detection. During the virus pandemic, there was no microscopic diagnosis performed. Individuals with any other respiratory condition, such as COPD or cystic fibrosis, were excluded from the study.

The medical records and/or the patient or his relative interview were used to gather the medical history and demographic data.

### Nucleic acid extraction and SARS-CoV-2 detection

The nasopharyngeal swabs were used to detect SARS-CoV-2. The total nucleic acid extraction was performed using the automated BIOBASE nucleic acid extraction kit employing the magnetic beads method (Biobase Biodustry [Shandong] co. ltd). Following extraction, the eluted nucleic acid samples were utilized to detect SARS-CoV-2. The SARS-CoV-2 reverse transcriptase-qPCR assay was performed according to the CDC methodology utilizing the TIANLONG: Real-time PCR System with 48-well block equipment and the LiliFTM COVID-19 Multi Real-time RT-PCR Kit. A SARS-CoV-2 assay result was classified as positive if the ribonuclease P (RNP) gene and either the N1 or N2 gene were detected, and as negative if only the RNP gene was identified. RNP was utilized to determine the sample quality and to detect PCR inhibition. Data related to primers and probe sequence for SARS-CoV-2, PCR conditions, as well as positive and negative controls are available and adapted from the previous studies [21,22].

### *P. jirovecii* detection

All sputum samples were tested for *P. jirovecii*. Beta-tubulin (BT) and dihydrofolate reductase (DHFR) genes were the directed targets of *P. jirovecii*. These sputum samples were collected 10 days after the onset of COVID-19 symptoms.

*P. jirovecii* purified DNA templates of cell-conditioned medium from cultured primary epithelial cells were used for real-time qPCR of the calibration data. All real-time qPCR assays were TaqMan assays. Table 1 lists the primers and probes sequences (Eurofins, UK). Ten-fold serial dilutions of the four concentrations of the standard DNA (1000, 100, 10, and 1 copies/μL) with five replicates were performed to make the standard curve for *P. jirovecii*. The amplification reaction of the five replicates was conducted for each of the four concentrations of the standard DNA using 90 μL (10-fold serial dilutions) of a specific type of buffer (a “blocking” nucleic acid; lambda DNA, yeast tRNA, salmon sperm DNA) (Sigma-Aldrich, UK).

**TABLE 1:**
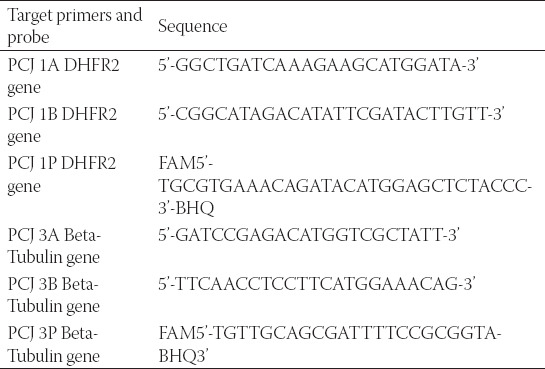
Primer and probe sequences of *Pneumocystis jirovecii* targets

We used 9 μL of master-mix (Roche Diagnostics, UK) (Table 2) and 6 μL *P. jirovecii* DNA template for each replicate of the four concentrations, and this final step was done in a duplicate so a total of 40 wells, for each target gene (both BT and DHFR genes), each with 15 μL. We used four negative control samples for each target gene.

**TABLE 2:**
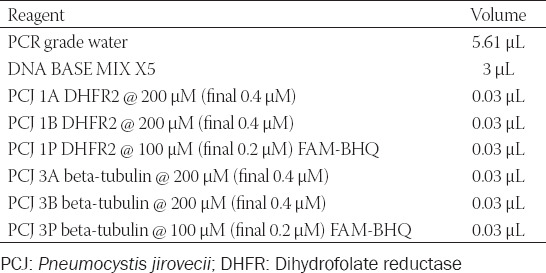
Master mix preparation for *Pneumocystis jirovecii*

The absolute quantification was used to analyze the samples in a similar principle of a previous recent publication [23]. For Ct values ≤40 cycles, a positive result was recorded, whereas a negative result was recorded in the lack of a Ct value and for Ct values greater than 40 cycles. In addition, a positive RNP value with Ct ≤37 cycles was required. When RNP levels exceed 37 cycles, the sample is frozen, thawed, re-extracted, and re-tested. For the second test, samples with RNP values larger than 37 cycles would be excluded from analysis due to either low quality or the presence of an inhibitor of the PCR.

Six microliters from each patient’ specimen were pipetted into the 48 wells-plate after dispensing 9 mL of the master-mix. Plates were sealed using sealing foil, then centrifuged at 1500 rotations per minute and placed in the TIANLONG: Real-Time PCR System. The TaqMan PCR program consisted of 50°C for 15 min, 95°C for 5 min, 45 cycles of 95°C for 10 s, and then 60 °C for 1 min, with a final step of 40°C for 15 s.

### Ethical statement

The approval of the study was gained from the Research Ethics Board of Applied Science Private University, Amman, Jordan (2021-PHA-35) and from Al-Rayhan Medical Center (2021-IRB-9-2). Informed consent was obtained from every patient.

### Statistical analysis

Data were analyzed using SPSS Statistics version 24 (IBM Corporation, USA). All data are presented as mean (standard deviation) or number (percentage).

## RESULTS

### Study population and characteristics

During the study period and among patients with asthma and COVID-19, 31 had a sputum sample for *P. jirovecii* analysis. Patients’ demographic data and clinical variables are described in Table 3. The patients mean age (SD) was 45.35 (12.020). Among those 31 asthmatic patients, 19 (61.3%) were female. Overall, 4 (12.9%) have diabetes mellitus. Around three-quarters were non-smokers (77.4%). More than one-half of the included participants were second-hand smokers (64.5%). The majority (90.3%) of the included asthmatic patients did not take any dose of the COVID-19 vaccine at the time of enrolment. The inhaled corticosteroid/long-acting beta-2 agonist was the used medication for all of the participants. During the study period, supplemental oxygen was used for 17 patients (54.8%) (Table 3). None of the study participants required hospitalization during the 14 days follow-up as all of the included patients were classified to have moderate/non-severe COVID-19 with non-severe pneumonia according to the World Health Organization classification [24,25]. The most frequently reported symptoms at the initial visit were cough and fever, as reported in all of the patients (100.0%) (Table 4).

**TABLE 3:**
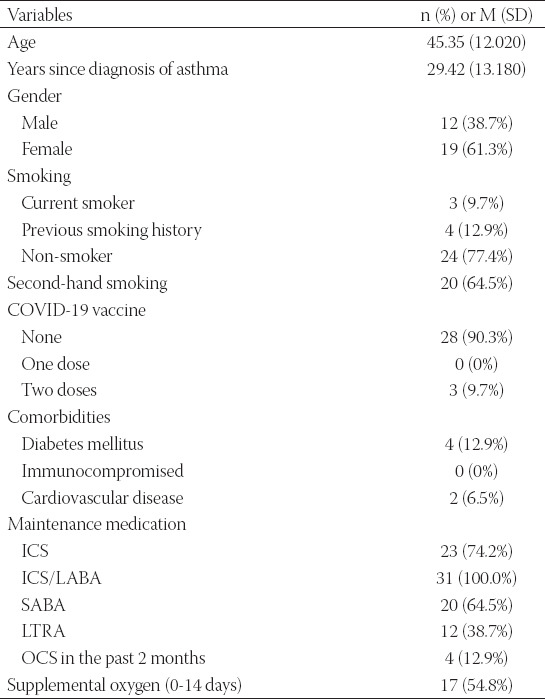
Demographics and clinical variables for the study participants (n=31)

**TABLE 4:**
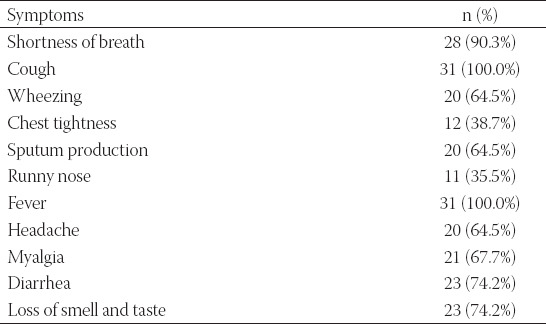
Presented complains at the initial visit

Three patients (9.7%) had a positive *P. jirovecii* qPCR. All of the three patients had used OCS in the past 2 months due asthma exacerbation. Diabetes was found in two of the three patients. Furthermore, OCS for COVID-19 was administered to these three patients. None of the included patients required mechanical ventilation and admission to the ICU. One patient died within 2 months of his COVID-19 diagnosis. Trimethoprim-sulfamethoxazole or other alternatives were not used to treat any of the patients. All of the patients were treated with broad-spectrum antimicrobial agents.

### *P. jirovecii* calibration data

The means of the C_t_ values with standard deviation were calculated for all the concentrations and replicates of *P. jirovecii* calibration data (Table 5). The mean (SD) of the correlation coefficient (r^2^) was −0.998 (0.001) for BT, and −0.996 (0.001) for DHFR targets.

**TABLE 5:**
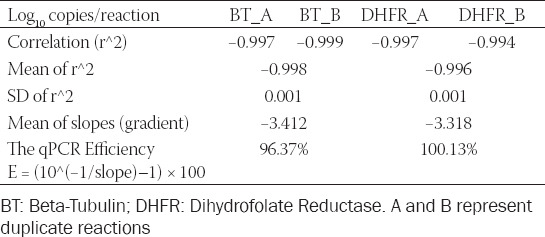
*Pneumocystis jirovecii* calibration data

**FIGURE 1:**
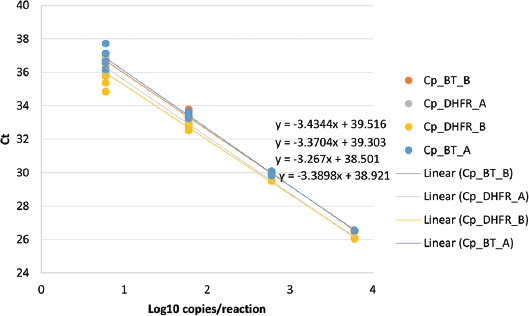
*Pneumocystis jirovecii* Calibration Curve. A and B represent duplicate.

By plotting the log of target DNA concentrations against C_t_ values, the standard curve was created; linear regressions from each of the replicate dilution series were used. Figure 1 depicts the calibration curves for *P. jirovecii*. The amplification efficiency (E) of the absolute quantification is the mean efficiency derived by E = (10^-Slope^–1) × 100. The results showed that the mean PCR efficiencies of BT and DHFR were 96.37% and 100.13%, respectively.

## DISCUSSION

The primary objective of this study was to ascertain the prevalence of *P. jirovecii* in Jordanian asthmatic patients following SARS-CoV-2 infection. We identified three cases of *P*. *jirovecii* using qPCR in sputum samples from 31 (9.7%) COVID-19 asthmatic patients. Concerning risk factors, all of these three patients used OCS in the preceding 10-14 days for COVID-19 and asthma exacerbation treatment. Two patients had a favorable prognosis, but one died within 2 months of the COVID-19 incident.

*P. jirovecii* is an opportunistic microorganism that is most prevalent in immunocompromised persons. Even though PCP was formerly linked with severe HIV infection, it, now, affects individuals who are immunocompromised for other reasons, including those who require corticosteroids [26,27].

The prevalence of positive *P. jirovecii* qPCR in patients with COVID-19 is unknown. A recent study reported positive *P. jirovecii* qPCR in 2/145 (1.4%) COVID-19 patients in ICU [16]. Another study discovered a detection of *P. jirovecii* PCR in 10/108 (9.3 %) of COVID-19 patients presenting with ARDS [13]. Numerous cases of SARS-CoV-2 coinfection with *P*. *jirovecii* have also been recorded in patients with recognized risk factors for PCP [1,15,16,28-31]. In one example, a patient who had been diagnosed with COVID-19 and treated with tocilizumab and glucocorticoids was later diagnosed with PCP. This raised the hypothesis, similar to this study, that immunomodulatory treatment for COVID-19 may have contributed to the development of PCP [31].

The diagnosis of PCP in COVID-19 cases, as well as the distinction between infection and colonization, are extremely difficult to make [16]. COVID-19 patients in the ICU may develop PCP as a result of mechanical ventilation, corticosteroid medication, or the presence of a cytokine storm [1]. However, radiographic similarities exist between the two illnesses, with the appearance of cysts or tiny reticular alterations on computed tomography (CT) scan indicating pneumocystosis [16,28,29]. It is possible that overdiagnosis of PCP in colonized patients will occur as a result of the high sensitivity of PCR, and distinguishing colonization from PCP can be difficult, particularly in immunocompetent patients [13,16,29]. In most cases, no direct examination is performed on patients with COVID-19 infection. In our study, we did not use staining due to the estimated risk of aerosolization during COVID-19. However, staining is quite useful for visualizing cysts or trophozoite forms and, therefore, for differentiating infection from colonization. This could be critical in determining the presence of these coinfections accurately.

Due to the high negative predictive value of the serum fungal marker (1→3)-β-D-Glucan (BG) assay, it may be used to rule out infection in some cases [13,32]. However, confirmation of the diagnosis requires further testing and well-matched clinical features. In addition to mycological criteria, the diagnosis of infection must be based on a number of other factors, including clinical deterioration, immunosuppression, severe lymphocytopenia, serum BG, and lactate dehydrogenase assays, and response to therapy [16]. PCP and moderate-to-severe COVID-19 have a number of clinical characteristics in common, making it difficult to distinguish between the two diseases. Both conditions are characterized by the presence of fever, cough, and hypoxia [33] and the presence of a wide range of radiographic findings, including diffuse ground-glass opacities [34]. The similarities between the two infections may be due to shared pathogenic mechanisms and interactions with pulmonary surfactant [35], which have been hypothesized. As a result of these and other similar findings, PCP is increasingly being recognized as a COVID-19 mimicker in critically ill patients [14].

A single-center case series included five patients diagnosed with PCP, one of whom had classical risk factors for PCP and the others were immunocompetent before the onset of COVID-19. Surprisingly, none of the patients with confirmed that PCP had detectable BG in repeated serum samples; these findings were explained by the fact that, while serum BG has a high negative predictive value for PCP diagnosis, its role is primarily recognized in HIV patients, and its sensitivity may be lower in immunocompetent hosts [36]. Each patient received a minimum of 2 weeks of high-dose corticosteroid. This is the most likely risk factor for PCP development in this cohort, along with CD4+ lymphopenia, which has been observed in the majority of patients affected by COVID-19 and is associated with a poor prognosis, particularly in younger patients [37,38].

Concurrent infection with SARS-CoV-2 and *P jirovecii* can create diagnostic difficulties. While most hospital laboratories now offer COVID-19 testing using nasopharyngeal swabs with a rapid turnaround time [39,40], PCP is more difficult to diagnose. Due to its increased sensitivity, in patients with severe hypoxia, bronchial alveolar lavage (BAL) fluid stays the standard method for PCP diagnosis [41]; however, doing a bronchoscopy to collect, a BAL specimen is an invasive procedure that is not always plausible in severely hypoxic patients and due to the possibility of SARS-CoV-2 aerosolization, greater procedural vigilance is required.

Corticosteroids for severe COVID-19 would, further, defer identification of cooccurring PCP, as these individuals may possibly improve temporarily as a result of steroids’ documented beneficial effect on severe PCP. Tocilizumab, under investigation as a possible COVID-19 treatment, has been related with PCP in the management of rheumatoid arthritis [42]. Accordingly, health-care professionals should be aware of the possibility of PCP.

PCP has a more subacute course than COVID-19 [43-45]. In comparison to COVID-19, PCP symptoms might continue many weeks before a diagnosis is made, with a median duration to diagnosis of around 1 week from disease beginning [2,46].

The part of the purpose of this research was to evaluate a method of TaqMan qPCR assay to detect *P. jirovecii*, from sputum samples of asthma patients infected with SARS-CoV-2. The findings of the assay targeting *P. jirovecii* were highly linear over the replicated dilution series (as observed from r^2^ values) and the data from independent replicates were highly reproducible. The use of the nested PCR in most of the previous studies and the high chance of contamination associated with the nested PCR may contribute to the inconsistent *P. jirovecii* prevalence. Whereas the assay in this study uses TaqMan real-time qPCR chemistry, so accurate detection and quantification of the microbial targets are more likely, as appropriate calibrations are available. Real-time qPCR avoids the cross-contamination and allows target quantification, unlike the nested PCR assay. In a multicenter study, in which four laboratories involved, it was demonstrated that there is a high risk of contamination with the nested PCR [47]. Using the rapid molecular methods, such as qPCR, should increase the yield of microbial detection and provide a better insight of the microbial association with asthma exacerbation [48,49]. The qPCR can detect small amounts of nucleic acids from the target pathogens; it does not rely on the viability of the pathogen which is probably less affected by previous antibiotic treatment compared with the culture-based approaches [50].

Although the findings of this study did not confirm a significant association of *P. jirovecii* and asthma exacerbation compared with the baseline, it could not be ruled out that *P. jirovecii* continued in the lower respiratory tract (LRT) from a preceding exacerbation. Further, work needs to be performed to solve the debatable issue whether *P. jirovecii* recovered from the LRT of patients with asthma indicate colonization, infection, or persistence. The previous research has established a link between *P. jirovecii* colonization and chronic lung illnesses such as asthma [19]. Patients with stable asthma show a greater enrichment of *P. jirovecii* in BAL fluid [51] and higher sera titers against whole cell *P. jirovecii* antigens [19], implying that they are more exposed to *P. jirovecii* than healthy persons. However, there are a number of *P. jirovecii* antigens that are highly immunogenic but do not confer protection against infection [52,53].

Our study is not without limitations. To begin, only 31 sputum specimens from COVID-19 patients were examined for *P. jirovecii* coinfection; however, all of the patients included had asthma, hence increasing the statistical power. PCP is difficult to diagnose in COVID-19, based on a single *P. jirovecii* qPCR result and the lack of additional tests to confirm coinfections in this study can be justified by the limitations of the other tests, especially during the pandemic and lack of a strong recommendation of other test for non-HIV patients. HIV markers were not provided for each patient, because HIV test is not commonly done in Jordan nor accepted by the patients. However, none of the included patients were with suspected HIV clinically. Finally, one patient in our study had a very poor prognosis, but it is unknown if this death is attributable to *P. jirovecii* or if the presence of *P. jirovecii* is only a marker of immunosuppression and severe form of COVID-19 infection.

Our research adds geographical data on the discovery of *P. jirovecii* in individuals with asthma and COVID-19. These coinfections are uncommon but substantial; consequently, PCP should be investigated in patients with moderate-severe COVID-19 who experience deterioration of their disease. Additional research is needed to elucidate the epidemiology of *P. jirovecii* detection in asthmatic and COVID-19 patients, in addition to the identification and management of this pathogen.

## CONCLUSION

This is the first study based in Jordan to demonstrate that *P. jirovecii* detection and COVID-19 can coexist and that it is important to maintain a broad differential diagnosis, especially in immunocompromised patients.

COVID-19 and PCP share some characteristics that may make identification more difficult. As a result, further diagnostic testing should be done in individuals with COVID-19 who have clinical data compatible with PCP and a possible coinfection. This is especially true for immunocompromised individuals.

An important risk factor for PCP development in COVID-19 patients is the use of corticosteroids for asthma exacerbations and/or the presence of COVID-19, and further study is needed to identify these risk factors and predictors of PCP development and to find the best preventative and therapeutics strategy.
